# Relations of self-regulation and self-efficacy for exercise and eating and BMI change: A field investigation

**DOI:** 10.1186/1751-0759-4-10

**Published:** 2010-09-03

**Authors:** James J Annesi, Srinivasa Gorjala

**Affiliations:** 1 YMCA of Metropolitan Atlanta, 100 Edgewood Avenue NE, Suite 1100, Atlanta, Georgia, USA; 2 Southern Regional Health Systems, Bariatric Center, 33 Upper Riverdale Road SW, Suite 121, Riverdale, Georgia, USA

## Abstract

**Objectives:**

This study aimed to assess relations of self-regulatory skill use with self-efficacy for exercise and appropriate eating, and the resulting change in weight associated with participation in a nutrition and exercise treatment supported by cognitive-behavioral methods.

**Methods:**

Adults with severe obesity (N = 95; mean BMI = 40.5 ± 3.9 kg/m^2^) participated in a 6-month exercise and nutrition treatment emphasizing self-regulatory skills. Changes in self-regulatory skills usage, self-efficacy, overall mood, and BMI were measured. Relations of changes in self-regulatory skill use and self-efficacy, for both physical activity and appropriate eating, were assessed, as was the possibility of mood change being a mediator of these relationships. Indirect effects of the variables associated with the present treatment on BMI change were then estimated.

**Results:**

For both exercise and appropriate eating, changes in self-regulation were associated with self-efficacy change. Mood change partially mediated the relationship between changes in self-regulation for appropriate eating and self-efficacy for appropriate eating. Self-efficacy changes for physical activity and controlled eating, together, explained a significant portion of the variance in BMI change (R^2 ^= 0.26, *p *< 0.001). The total indirect effect of the treatment on BMI change was 0.20.

**Conclusion:**

Findings suggest that training in self-regulation for exercise and eating may benefit self-efficacy and weight-loss outcomes. Thus, these variables should be considered in both the theory and behavioral treatment of obesity.

## Background

Results of standard weight-loss treatments have been disappointing [1]. Although regular exercise and appropriate eating will manage weight, individuals are not typically able to successfully negotiate common barriers such as time pressures, discomfort, and social pressures, over time, and typically regain any weight lost in short order [2]. This pattern is so typical that treatments are usually evaluated by percentage of weight regained, rather that if weight loss is maintained [3]. Because of poor prospects with such approaches, surgeries such as gastrointestinal bypass, vertical banded gastroplasty, and laparoscopic gastric banding are increasingly the weight-loss treatments of choice for those with class 3 obesity (body mass index [BMI] ≥ 40.0 kg/m^2^) and class 2 obesity (BMI 35.0-39.9 kg/m^2^) with one or more comorbidities [4]. Although behavioral weight loss treatments have often been atheoretical, and dependent largely on persons responding to information on appropriate eating and exercise (where there is little evidence of success), accepted behavioral theories have sometimes been used as a basis for intervention strategies [5].

Self-efficacy theory, for example, posits that people's judgments of their capabilities to carry out actions will predict their behaviors, and suggests that self-efficacy may be increased through attainment of prior success, imitating others' performance, verbal and social persuasion, and perceptions that positive psychological states may be achieved [6]. Researchers posit that self-efficacy is a precondition of behavior change [7]. Although increases in self-efficacy have been attempted through such general methods as encouragement and observation of the success of others, it is possible that purposefully fostering self-regulatory skills use (eg, cognitive restructuring, stimulus control) early in a treatment will induce increased self-efficacy over time. It has been suggested that "behavioral self-regulation" [[8], p 545] and "coping responses" [[9], p 38] will lead to self-efficacy and improved outcomes because of their direct connection with overcoming barriers and establishing feelings of competence and success. Because low mood has been shown to be associated with reduced use of self-regulatory skills and perceptions of self-efficacy [10], and positive changes in mood may promote a "healthier psychological climate" that enhances confidence in pursuing weight management behaviors [[11], p 320], the established effects of exercise program participation on improved mood [12] may mediate (significantly reduce or cancel out the relationship between two variables by its entry) this relationship. It has been suggested that treatments should first be based on accepted theory, and then their effects decomposed to determine if changes in variables consistent with the theory predicted outcomes as expected [13]. Such a thorough analytic approach has often been lacking in weight-loss research.

Thus, the present investigation aimed to test a treatment of exercise and nutritional support, based on tenets of self-efficacy theory, which emphasized self-regulatory skills. It was thought that the treatment would be associated with significant increases in measures of self-regulatory skills usage, self-efficacy, and mood. More specifically, changes in self-regulatory skills were expected to be associated with changes in self-efficacy for both physical activity and appropriate eating, but be significantly mediated by mood changes. Additionally, a significant portion of the variance in BMI change was expected to be accounted for by changes in self-efficacy for physical activity and appropriate eating.

Formerly sedentary adults with class 2 and 3 obesity were selected for participation because of the considerable need for a better understanding of theory-based psychological predictors of weight loss in these highly at-risk subgroups. It was hoped that increased knowledge of the effect of self-regulatory skills usage on self-efficacy would yield important data for weight management theory and application.

## Methods

### Subjects

Men and women responded to advertisements in local newspapers for participation in research incorporating physical activity and nutrition instruction for weight loss. Requirements were age ≥ 21 years, weight of BMI 35-50 kg/m^2 ^(class 2 and class 3 obesity), and no regular exercise (< 20 minutes/week reported) in the previous year. Pregnancy or use of medication for weight loss or a diagnosed psychological/psychiatric condition was cause for exclusion. A written statement of adequate physical health to participate was required from a physician. Institutional review board approval and written consent from all participants was obtained. The 72 (76%) women and 23 (24%) men (mean age = 43.5 ± 10.0 years; mean BMI = 40.5 ± 3.9 kg/m^2^) initiated the treatment at a YMCA facility in the southeast US. The racial make-up was 54% White, 43% African-American, and 3% of other racial groups. Based on reported addresses and the most recent census data, 89% were in the lower-middle to middle classes.

### Measures

#### Self-regulatory skill usage

Self-regulation skills usage for both physical activity (Self-reg-PA) and appropriate eating (Self-reg-EAT) was measured using adapted versions of a scale by Saelens et al. [14], where items are based on intervention content. Thus, the items for the present two scales were based on self-regulatory skills for eating and self-regulatory skills for physical activity addressed within the present treatment. Each scale required responses to 10 items ranging from 1 (Never) to 5 (Often). Examples were, "I keep a record of my physical activities" (Self-reg-PA) and "I say positive things to myself about eating well" (Self-reg-EAT). Internal consistency (alpha value = 0.75), test-retest reliability over 2 weeks (0.77), and predictive validity, were supported [15]. For the present versions, alpha coefficients were 0.79 and 0.81, respectively; and the test-retest reliabilities were 0.78 and 0.74, respectively, in pilot research.

#### Self-efficacy

The Exercise Self-Efficacy Scale (ESE) [16] measured perceived competence to overcome barriers to completing exercise. Responses to its 5 items that begin with the stem, "I am confident I can participate in regular exercise when:" (eg, "I have more enjoyable things to do"), range from 1 (Not at all confident) to 7 (Very confident). Alpha coefficients were reported to be 0.82 and 0.76, and test-retest reliability over 2 weeks was 0.90 [17]. The alpha value for the present sample was 0.77.

The Weight Efficacy Lifestyle Scale (WEL) [18] measured self-efficacy for appropriate eating. It has 5 subscales (ie, Negative Emotions, Availability, Social Pressure, Physical Discomfort, Positive Activities) of 4 items each (eg, "I can resist eating even when high-calorie foods are available") that are summed for a total score. Item responses range from 0 (Not confident) to 9 (Very confident). Alpha coefficients were reported to range from 0.70-0.90 [18], and was 0.83 for the present sample.

#### Mood

Total Mood Disturbance (TMD) is derived by aggregating response scores from the 6 subscales (ie, Depression, Tension, Fatigue, Vigor, Confusion, Anger) of the Profile of Mood States Short Form [19]. Respondents rate feelings "over the past week" on 30 items (5 for each subscale) ranging from 0 (Not at all) to 4 (Extremely). Alpha coefficients were reported to range from 0.84-0.95, and test-retest reliability at 3 weeks averaged 0.69 [19]. For the present sample, alpha values ranged from 0.79-0.92.

#### BMI

A stadiometer and recently calibrated digital scale were used to measure BMI (kg/m^2^).

Change scores on all measures were calculated by subtracting the baseline score from the score at week 12 (self-regulatory measures) and the baseline score from the score at week 24 (all other measures).

### Procedure

Participants received access to a YMCA wellness center and were enrolled in a nutrition and exercise treatment based on tenets of self-efficacy theory. The exercise support portion of the treatment consisted of 6 one-on-one meetings of 45-60 minutes each, with a trained wellness specialist over 6 months (5 meetings in the initial 3 months, with the final meeting being a review), conducted primarily in a private office and supported by a computer program [20]. Instruction in an array of self-regulatory methods (eg, long- and short-term goal setting, annotating incremental progress, thought-stopping, cognitive restructuring, stimulus control, self-reward, preparing for specific types of barriers, recovery from lapses) was the primary focus of the initial 12 weeks. An orientation to available exercise equipment and facilities was also given. Cardiovascular exercise plans were based on each subject's preference and tolerance, but uniformly progressed from 20 minutes at a light-moderate to moderate intensity 3-4 days per week [21].

The nutrition portion of the treatment consisted of 6 1-hour sessions over the initial 3 months [22]. They were lead by a wellness specialist in a group format of approximately 15 subjects. Examples of program components were (1) understanding macronutrients, (2) using the US Food Guide Pyramid, (3) developing a plan for meals and snacks, and (4) use of self-regulation methods. The self-regulation skills taught were similar to those in the exercise component, but focused on managing eating behaviors.

Wellness specialists were blind to the purposes of the investigation. For both the nutrition and exercise segments of the treatment, the development of self-regulatory skills and self-efficacy was emphasized. Compliance with treatment protocols was assessed by YMCA wellness administrators under the direction of a study investigator. Assessments were administered in a private area at baseline, week 12, and week 24.

### Data analyses

An intention-to-treat design was used that retained data from all subjects who initiated treatment. Multiple imputation [23] was used for the 26% of overall missing cases. An a priori power analysis for multiple regression indicated that to detect a moderate effect size (f^2 ^= 0.15) at the statistical power of 0.90, a minimum of 87 subjects was required. An a priori alpha level of 0.01 (2-tailed) was set to adjust for multiple tests.

Initially, within-subject changes over 12 weeks in Self-reg-PA and Self-reg-EAT, and over 24 weeks in ESE, WEL, TMD, and BMI, associated with the treatment, were assessed by dependent t tests. The shorter time frame for changes in the self-regulation measures was based on completion of the self-regulation skills portion of the training by 12 weeks after initiation. Consistent with previous research in field settings [24], actual score changes, rather than changes controlling for baseline scores or percent changes, were incorporated to best account for the naturally occurring range of baseline values found in the present sample type. Skewness and kurtosis of change scores were assessed where, as suggested [25], values within 3 and 10 SE, respectively, represented an approximately normal distribution.

Using linear regression analysis, the relationships of change in Self-reg-PA with ESE change, and Self-reg-EAT change with change in WEL, were derived. A multiple regression equation with changes in ESE and WEL as predictors of BMI change was next calculated. The conservative Sobel test [26], which requires normality in the distribution of variables, was employed using the Baron and Kenny method for assessing mediation [27] to indicate if change in TMD significantly mediated the relationships of Self-reg-PA change with ESE change, and change in Self-reg-EAT with WEL change.

To derive the total indirect effect of the treatment on BMI change, the relationships of the treatment with changes in Self-reg-PA and Self-reg-EAT were first estimated through conversions of the associated dependent t-test values: r = √t^2^/t^2 ^+ df. Then, individual paths from treatment to BMI change were summed [28,29].

## Results

All within-subject changes were significant. Change scores of measures of all predictor variables were approximately normally distributed and displayed in Table 1.

**Table 1:** Within-subject changes and distributions of changes in self-regulation, self-efficacy, and mood measures, and BMI (N = 95).

					t_94 _	d	95% CI	Change scores		**Skewness**^**a**^	**Kurtosis**^**b**^
	Mean	SD	Mean	SD				Mean	SD		
	Baseline		Week 12								
Self-reg-PA	20.05	5.49	26.03	8.03	7.29**	1.09	4.35, 7.61	5.98	7.99	0.64	0.01
Self-reg-EAT	21.06	5.87	25.16	6.95	7.20**	0.70	2.97, 5.22	4.09	5.54	0.70	0.10
	Baseline		Week 24								
ESE	30.45	12.41	33.57	11.19	3.04*	0.25	1.08, 5.15	3.12	10.00	-0.03	1.04
WEL	101.91	34.27	118.61	34.67	5.27**	0.49	10.41, 23.00	16.71	30.91	0.68	0.87
TMD	20.70	16.10	12.47	18.70	5.72**	-0.49	-11.08, -5.37	-8.23	14.03	-0.70	- 0.09
BMI (kg/m^2^)	40.49	3.91	39.29	4.07	6.52**	-0.30	-1.56, -0.83	-1.19	1.79 -	1.84	3.41

Self-reg-PA = self-regulation for physical activity; Self-reg-EAT = self-regulation for appropriate eating; ESE = Exercise Self-Efficacy Scale; WEL = Weight Efficacy Lifestyle Questionnaire; TMD = Total Mood Disturbance; BMI = body mass index.

d = Cohen's measure of effect size.

^a^For skewness, SE = 0.25

^b^For kurtosis, SE = 0.49

*p < 0.01 **p < 0.001

**Figure 1:**
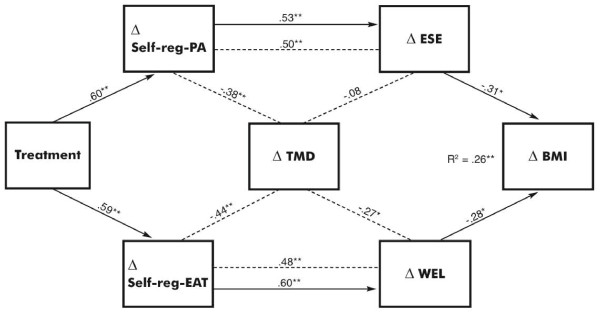
**Relationships of changes in measures of self-regulatory skills use, self-efficacy, mood, and BMI**. Self-reg-PA = self-regulation for physical activity; Self-reg-EAT = self-regulation for appropriate eating; ESE = Exercise Self-Efficacy Scale; WEL = Weight Efficacy Lifestyle Questionnaire; TMD = Total Mood Disturbance; BMI = body mass index. Δ = change from baseline to week 12 (Self-reg-PA, Self-reg-EAT), and baseline to week 24 (ESE, WEL, TMD, BMI). *p < 0.01 **p < 0.001.

Relationships between changes in Self-reg-PA and ESE, and Self-reg-EAT and WEL were significant (Figure 1). Only the latter relationship was partially mediated by TMD changes, Z = 2.57, *p *< 0.01. Approximately 26% of the variance in BMI change was explained by changes in ESE and WEL, which was significant, F_2,92 _= 16.05, *p *< 0.001. Both corresponding β-values were also significant (Figure 1). Relationships of the treatment with changes in Self-reg-PA and Self-reg-EAT were significant (Figure 1). The total indirect effect of the treatment on BMI change was 0.20.

## Discussion

As expected, the behavioral treatment emphasizing self-regulatory skills and exercise from treatment outset was associated with significant within-subject improvements in self-regulatory skill use, self-efficacy, and mood. Also as expected, increases in self-regulatory skill use over 3 months predicted increased self-efficacy over 6 months for both exercise and appropriate eating. As hypothesized, mood change had a significant relationship with changes self-regulation for appropriate eating, self-efficacy for appropriate eating, and their interrelationship, which manifested as significant partial mediation. Such relationships did not occur with the corresponding analyses for exercise.

Extensions of this research should seek to determine if mood decrements induce a propensity for *acute episodes *of low mood, and thus may reduce self-regulation and confidence in controlled eating ("emotional eating"), or whether longer-term negative mood is, alone, associated with self-regulation decrements and reduction in self-efficacy. Whether exercise program-induced *improvements *in mood *enhance *self-regulation and confidence for weight loss also requires consideration. Consistent with self-efficacy theory [6], perceptions of competence to overcome both barriers to physical activity and appropriate eating, together, explained a notable portion of the variance in BMI change. Thus, it makes sense to carefully attend to self-efficacy changes in future weight-loss treatment research, while additional psychological variables (eg, motivation, body image) that may lead to a more comprehensive prediction model of weight loss and maintenance should be investigated further.

Although of a somewhat brief duration with a volunteer sample, this study added to the minimal research on effects of behavioral weight-loss treatments for the severely obese through psychological pathways [30,31]. It extended earlier propositions of the contribution of exercise participation to weight loss [11]. It has been proposed that for obese and formerly sedentary persons, the positive effects of exercise program participation on weight loss are more from its positive psychological effects than the minimal caloric expenditure initially possible [24]. There was indirect support for this here. Replications of this research are required across types of subjects (eg, specific ethnicities, ages, weight classifications, and sex) and under different medical circumstances (eg, diabetes, cancer, post-bariatric surgery) to assess generalizability of findings. Inclusion of control conditions will also be needed. It is recommended that, although challenges to internal validity exist due to social support and expectation effects, field-based research is a priority because of its easily translatable findings leading to prompt practical application [32]. Future research should extend examination of the relationship between self-regulation and self-efficacy, especially in the area of their directionality or reciprocality, and seek to refine behavioral models to maximize sustained weight loss through innovative, evidence-based treatments.

## Conclusions

Cognitive-behavioral training in self-regulation may benefit self-efficacy for appropriate eating and exercise in obese adults seeking weight loss. Mood may partially mediate this relationship for eating. Exercise may promote weight loss through psychological pathways, and well beyond associated energy expenditures.

## Competing interests

The authors declare that they have no competing interests.

## Authors' contributions

JJA conceptualized the study, developed the design, and analyzed the data. SG conceptualized aspects of the study. Both authors read and approved the final manuscript.
